# Impact of persistent peripheral neuropathy on health-related quality of life among early-stage breast cancer survivors: a population-based cross-sectional study

**DOI:** 10.1007/s10549-022-06670-9

**Published:** 2022-08-09

**Authors:** Kristina Engvall, Henrik Gréen, Mats Fredrikson, Magnus Lagerlund, Freddi Lewin, Elisabeth Åvall-Lundqvist

**Affiliations:** 1 Department of Oncology, Region Jönköping County, SE-551 11 Jönköping, Sweden; 2 Department of Oncology and Department of Biomedical and Clinical Sciences, Linköping University, SE-581 85 Linköping, Sweden; 3 Division of Clinical Chemistry and Pharmacology, Department of Biomedical and Clinical Sciences, Linköping University, SE-581 85 Linköping, Sweden; 4 Department of Forensic Genetics and Forensic Toxicology, National Board of Forensic Medicine, SE-587 58 Linköping, Sweden; 5 Department of Biomedical and Clinical Sciences and Forum Östergötland, Linköping University, SE-581 85 Linköping, Sweden; 6 Department of Oncology, SE-391 26 Kalmar, Sweden

**Keywords:** Adjuvant therapy, Chemotherapy, Taxane, Chemotherapy-Induced Peripheral Neuropathy CIPN, Taxane-induced peripheral neuropathy, Survivorship, Breast cancer survivorship, QLQ-C30, CIPN20, Quality of life, Health-related quality of life, Functional health, Financial toxicity

## Abstract

**Background:**

We explored the impact of persistent sensory and motor taxane-induced peripheral neuropathy (TIPN) symptoms on health-related quality of life (HRQL) among early-stage breast cancer survivors (ESBCS).

**Methods:**

A population-based cohort of 884 residual-free ESBCS received a postal questionnaire, including the EORTC chemotherapy-induced PN (CIPN20) and the EORTC QLQ-C30 instruments. Mean scores of QLQ-C30 scales among ESBCS with and without TIPN were calculated and adjusted for confounding factors (age, lifestyle factors, co-morbidities; linear regression analyses). Interpretation of QLQ-C30 results were based on guidelines.

**Results:**

Response rate was 79%, and 646 survivors were included in the analysis. In median, 3.6 (1.5–7.3) years had elapsed post-taxane treatment. All TIPN symptoms had a significant impact on global QoL, which worsened with increased severity of TIPN. Between 29.5% and 93.3% of ESBCS with moderate-severe TIPN reported a clinical important impairment of functioning and personal finances, 64.3–85.7% reporting “difficulty walking because of foot drop,” and 53.1–81.3% reporting “problems standing/walking because of difficulty feeling ground under feet” had impaired functioning/finances. The difference in mean scores between affected and non-affected survivors was highest for “numbness in toes/feet” and “difficulty walking because of foot drop.” Moderate-severe “difficulty climbing stairs or getting out of chair because of weakness of legs” and “problems standing/walking because of difficulty feeling ground under feet” were associated with the largest clinically important differences on all scales.

**Conclusion:**

Persistent sensory and motor TIPN is associated with clinically relevant impairment of global QoL, functioning, and personal finances among ESBCS, which increased with level of TIPN severity.

**Supplementary Information:**

The online version contains supplementary material available at 10.1007/s10549-022-06670-9.

## Background

Worldwide, breast cancer (BC) is the most prevalent cancer with 8 million of women alive (end of 2020) after being diagnosed with BC in the past 5 years [1]. A reduction of BC mortality rates has been related to advancement in screening, diagnostics, and therapeutics. However, diagnosis and treatment of BC may have a significant impact on well-being and daily life functioning [2, 3]. The treatment of early-stage breast cancer (ESBC) usually consists of surgery and radiotherapy, which are often combined with chemotherapy, hormonal antitumoral treatment, and targeted therapy, depending on the patient and tumor characteristics. Late side effects among survivors can therefore vary depending on the treatment exposure. Since more than a decade, taxanes have been a standard component of adjuvant chemotherapy for ESBC [4], a treatment associated with persistent taxane-induced peripheral neuropathy (TIPN) [5, 6]. Little is known about the impact of persistent TIPN on health-related quality of life (HRQL) of long-term ESBC survivors.

Patient-reported outcome measures (PROMs) are used for assessing HRQL in clinical trials [7, 8]. One of the most used and validated instruments in cancer studies is the European Organization for Research and Treatment of Cancer Quality of Life Questionnaire Core 30 (EORTC QLQ-C30). Several publications provide guidelines for interpretating differences in QoL scores [9, 10]. To facilitate the interpretation of differences in QLQ-C30 scores, Cock et al. combined systematic review of published studies on a wide range of cancers, expert opinions, and meta-analysis to estimate differences corresponding to trivial, small, medium, and large effects for each QLQ-C30 scale [11]. However, the perspectives of patients were lacking. Recently, the EORTC QoL group published thresholds for clinical importance (TCIs) for the functional and symptom scales of the QLQ-C30 [12]. In contrast to the guidelines by Cock et al., the study by Giesinger et al. was based on cancer patients’ views; data from 498 European cancer patients were analyzed. The TCIs may, in a research context, be used for the calculation of prevalence rates. The thresholds may also be used as reference values and increase the questionnaire’s application in clinical practice.

As previously reported, we performed a population-based cross-sectional cohort study on recurrence-free ESBC survivors treated up to seven years earlier with (neo)adjuvant taxane chemotherapy regimens to explore the risk for persistent TIPN [13]. We hypothesized that there is an excess risk for persistent neuropathy among long-term ESBC survivors as measured with the EORTC-CIPN20 instrument, compared with women without prior cancer. Our findings showed that the risk for 13 individual symptoms of sensory and motor PN was significantly higher among survivors compared to age- and residency-matched women without prior cancer, but the prevalence and severity varied by individual neuropathic symptom. The secondary objective of the study, the results of which we present here, was to explore whether these 13 individual sensory and motor symptoms of persistent TIPN had a clinically relevant impact on global QoL, functional health, and personal finances among ESBC survivors.

## Methods

### Study design

A cross-sectional cohort study of recurrent-free women with ESBC who had received taxane chemotherapy as part of primary treatment.

### Study population

**Fig. 1:**
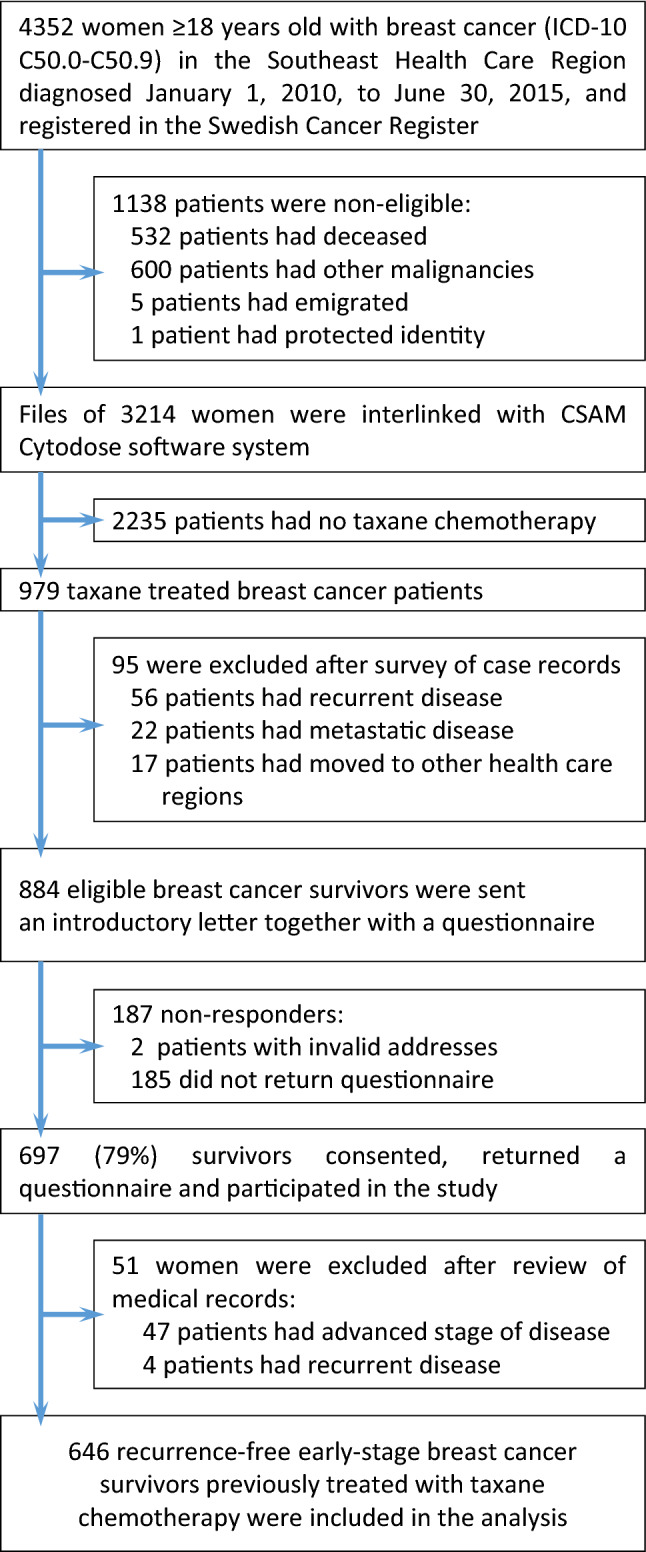
Flowchart of the study population showing eligible criteria, reasons for non-participation, and participation rate

We identified a cohort of 4352 women, at least 18 years old, and diagnosed with BC between 2010 and 2015 in the Southeast Health Care Region, Sweden. The eligibility criteria were early-stage disease without recurrence, no other malignancies, taxane-based chemotherapy as part of primary treatment, and not lost to follow-up. After excluding non-eligible women, 884 survivors remained (Fig. 1).

### Data collection

We constructed a study-specific questionnaire which included three validated instruments, i.e., the EORTC QLQ-C30, the Hospital Anxiety and Depression Scale (HADS) instrument, and the EORTC QLQ chemotherapy-induced peripheral neuropathy, CIPN20. Physical activity and alcohol consumption were assessed as specified by Olsson et al. [14] and Bush et al. [15]. Additional questions addressed, e.g., demographics, body mass index (BMI), co-morbidities, exogenous estrogens, menopausal status, lifestyle factors, chemotherapy-induced side effects, and their impact on specific daily life activities. The additional questions were tested for face validity. Adjustments of the questions were made successively until no further comments were made. The postal questionnaire study was conducted between September 2017 and January 2018.

Medical records were reviewed and data on tumor and treatment characteristics were recorded. Topographical codes C50.0–C50.9 from the International Classification of Diseases (ICD-10) and the TNM staging classification (7th Edition) for BC were used. Taxane regimens of docetaxel and paclitaxel were considered interchangeable in the guidelines in use and the choice made depended on local preferences. The predominant regimens were three courses of docetaxel 100 mg/m^2^ every 3 weeks and 12 courses of weekly paclitaxel 80 mg/m^2^. A detailed description of the study population and methods has previously been reported [13].

The EORTC QLQ-C30 (version 3.0) instrument was chosen as outcome measure to estimate the impact of self-reported symptoms of persistent PN on global health status (GHS)/QoL, functional health, and personal finances. The instrument comprises 30 items that can be summarized in 15 scales: GHS/QoL scale (two items); five functional scales, i.e., physical (PF, five items); role (RF, two items); emotional (EF, four items); cognitive (CF, two items) and social functioning (SF, two items); one item on financial difficulties, FI; and nine symptom scales (fatigue, nausea, vomiting, pain, dyspnea, insomnia, appetite loss, constipation, diarrhea). Data from the symptom scales are not reported here since these falls outside the scope of the study.

Each item of the QLQ-C30 questionnaires is measured on a four-point Likert scale ranging from “not at all” to “very much,” except for the GHS/QoL scale, which ranges from poor to excellent (1–7). Scores were linearly transformed to a 0–100 scale [16]; a higher score on the GHS/QoL scale and the functional scales means better GHS/QoL and functional health, whereas a higher score on FI means more financial difficulties. Missing items were imputed by the method advocated by the EORTC QLQ research group: if at least half of the items from a scale were completed, the mean value for these items was imputed for those missing.

### Statistical analysis

All pages from the questionnaire were scanned and transformed to Excel as previously described [13]. Different cut-off levels were used. ESBC survivors were dichotomized into not having the symptom or having the symptom (“a little,” “quite a bit,” or “very much”), in the past six months. The unadjusted mean scores (standard error of the mean, SE) of GHS/QoL, functional scales, and FI between ESBC survivors not having the symptom and those survivors affected by PN were calculated with Student’s *t* test. Linear regression analyses were performed to adjust for age, BMI, civil status, educational level, employment status, alcohol consumption, exercise, smoking, and co-morbidities. To explore the impact of moderate-severe PN on the same outcome measures, we combined the response categories “quite a bit” and “very much” and compared with survivors reporting no symptom or “a little.” The Bonferroni method was used to adjust for multiple comparisons within each symptom. Quantile (median) regression was used for test of trend of GHS/QoL among patients reporting different levels of PN symptoms and adjusted for age, BMI at survey, and treatment for diabetes mellitus. We also explored, by quantile regression, the impact of TIPN symptoms reported as “a little” versus not having the specific symptom at all after adjusting for the same confounding factors.

Published guidelines were used to define clinically important differences (CIDs) between ESBC survivors with and without PN [11]. The mean difference in scores was categorized into four groups depending on their estimated clinical relevance: a large difference was defined as one representing unequivocal clinical relevance; a median difference as clinically relevant but to a lesser extent; a small difference as clinically relevant but subtle; and a trivial difference as circumstances unlikely to have any clinical relevance. The estimates vary between scales, from nine points for a medium difference in CF to 19 points in RF. EF subscale was omitted due to the medium estimate being lower than the estimate for small effects [11]. We used the TCIs published by Giesinger et al. [12] as reference values when estimating the prevalence rate of affected and non-affected ESBC survivors with clinically important impairment of functional health and personal finances. For example, the TCI of PF is a mean score of 83, meaning that mean scores less than 83 indicate a clinically important impairment of PF. TCI for RF, SF, EF, and CF were 58, 58, 71, and 75, respectively. However, for symptom scales including FI, mean scores higher than the TCI (for FI 17) indicate a clinical important problem [12]. Comparisons in additional questions were tested by Pearson chi-square. Statistical analyses were performed using IBM SPSS version 26, except for the quantile regression that was performed with Stata version 16.1. Tests were two-sided and *p* values regarded significant if *p* < 0.05.

## Results

884 ESBC survivors were invited to participate in the study, 697 (79%) consented and filled in the study-specific questionnaire. After review of the medical records, 51 women were excluded due to non-eligibility. Hence, 646 ESBC survivors were included in the analysis (Fig. 1).

Clinical and treatment characteristics among ESBC survivors are presented in Table 1. At the time of the survey, the mean age was 61 years, most survivors (75.7%) were married or lived in partnership, 50.8% were employed, and 92.5% were postmenopausal. Most survivors were overweight, consumed alcohol, had never smoked, exercised at least 150 activity minutes per week, and reported co-morbidities of which musculoskeletal disorders dominated (52.9%). All except one woman had undergone breast surgery. Postoperative radiotherapy was delivered to 82.7%. Ongoing endocrine antitumoral treatment was received by 57.2% ESBC survivors. The median time from completed taxane treatment was 3.6 years (interquartile range 2.6–5.2 years).

**Table 1:** Demographic, clinical, and treatment characteristics

Characteristics	Breast cancer survivors *n* = 646
	No. (%)
Age, years^a^	
Mean (SD)	60.7 (11.2)
Median (min–max)	62.0 (31–86)
Marital status	
Married or in partnership	486 (75.2)
Unmarried or without partner	66 (10.2)
Divorced	55 (8.5)
Widow	35 (5.4)
Not reported	4
Highest level of education	
Elementary school	133 (20.6)
Secondary school	277 (42.9)
College/University	232 (35.9)
Not reported	4
Employment status	
Student	4 (0.6)
Unemployed	6 (0.9)
Employed, part-time	99 (15.3)
Employed, full-time	226 (35.0)
Disability pension	43 (6.7)
Retired	262 (40.6)
Not reported	6
Body mass index, BMI^a^	
< 18.5 (underweight)	11 (1.7)
18.5–24.9 (normal)	241 (37.3)
25–29.9 (overweight)	259 (40.1)
30–34.9 (obese)	91 (14.1)
> 35 (severely obese)	39 (6.1)
Mean (SD)	26.8 (4.8)
Not reported	9
Alcohol	
Problem consumption	68 (10.5)
Consumers	479 (74.2)
No consumption	99 (15.3)
Not reported	0
Smoking	
Current smoker	64 (9.9)
Former smoker	244 (37.8)
Never smoked	333 (51.6)
Not reported	5
Exercise weekly	
< 150 activity min/week	195 (30.2)
≥ 150 activity min/week	443 (68.6)
Not reported	8
Co-morbidities, self-reported^b^	
Musculoskeletal disorders	342 (52.9)
Cardiovascular disease	266 (41.2)
Neurological disease	88 (13.6)
Diabetes mellitus	38 (5.9)
Pulmonary disease	39 (6.0)
Menopausal status^a^	
Pre-menopausal	47 (7.3)
Post-menopausal	581 (89.9)
Not reported	18
Estrogen, exogenous (systemic or local)	
Yes	59 (9.1)
No	563 (87.2)
Not reported	24
TNM classification (7th edition)	
Tumor size	
T1	293 (45.4)
T2	311 (48.1)
T3	39 (6.0)
Not stated	3 (0.5)
Nodal status	
N0	258 (40.0)
N1	301 (46.6)
N2	87 (13.5)
Breast surgery	
Yes	645 (99.9)
Only axillary dissection	1 (0.2)
(Neo-)adjuvant chemotherapy	
Anthracycline-based regimens	644 (99.6)
Taxane-based regimens	646 (100)
Docetaxel	345 (53.4)
Paclitaxel	283 (43.8)
Alternating docetaxel and paclitaxel	18 (2.7)
Monoclonal antibody targeting HER2	
Trastuzumab	215 (33.3)
External beam radiotherapy	
Yes	534 (82.7)
No	112 (17.3)
Current endocrine antitumoral treatment^c^	
Tamoxifen	255 (39.5)
Aromatase inhibitor	114 (17.7)
GnRH analogues	14 (2.2)
Years since diagnosis	
Mean (SD)	4.5 (1.5)
Median (min–max)	4.1 (2.2–7.8)
Interquartile range (IQR)	3.1–5.7
Years since completed taxane treatment^d^	
Mean (SD)	3.9 (1.5)
Median (min–max)	3.6 (1.5–7.3)
Interquartile range (IQR)	2.6 -5.2

*GnRH* Gonadotropin-releasing hormone, *SD* Standard deviation

^a^At the time the questionnaire was completed

^b^Non-exclusive, i.e., several options were possible

^c^Self-reported data

^d^Number of years between last day of taxane treatment and completing the questionnaire

### Impact of TIPN on GHS/QoL

**Fig. 2:**
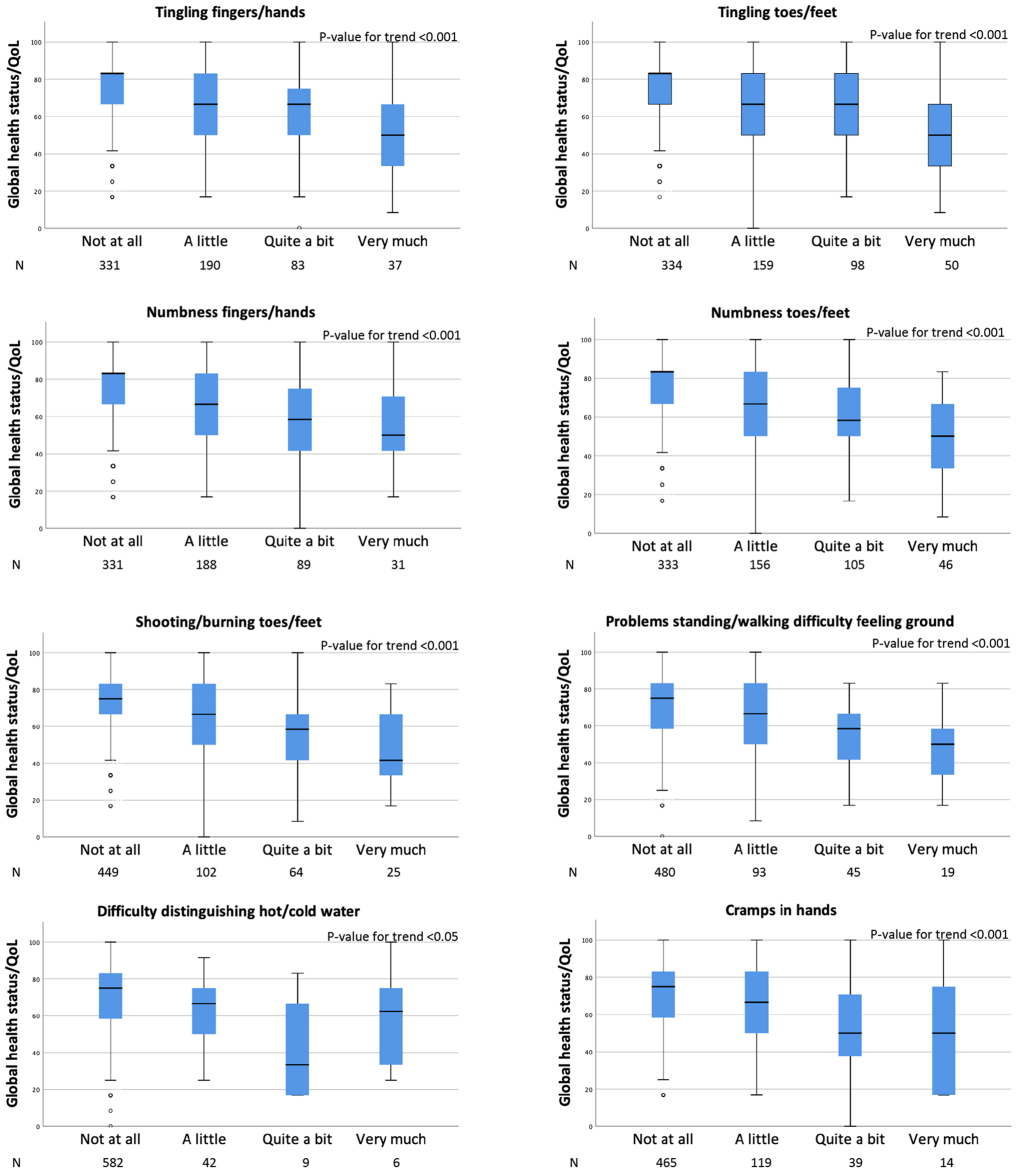

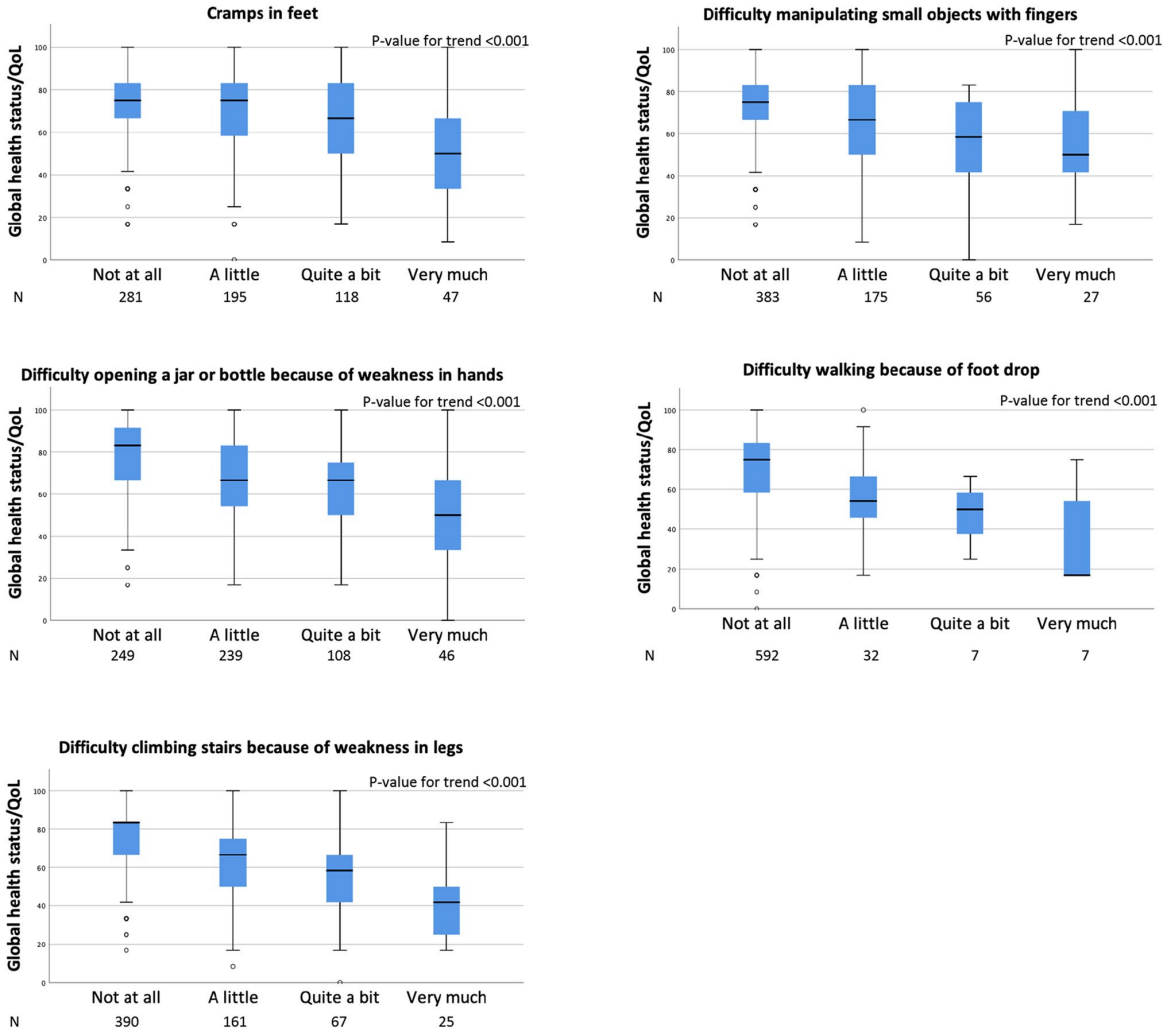
The impact of different levels of peripheral neuropathy symptoms among early-stage breast cancer survivors, illustrating worse GHS/QoL with increased severity of the symptom. The box expresses 25th, 50th (median), and 75th percentiles. The bar represents minimum and maximum values, and the dots represent observations considered to be outliers. The test for trend was done with quantile (median) regression and adjusted for age, BMI at survey, and treatment for diabetes mellitus

All 13 individual symptoms of persistent TIPN had a significant impact on GHS/QoL which worsened with increased severity of TIPN (Fig. 2). The significance level of *p*_trend_ < 0.001 was reached for all but one symptom, i.e., “difficulty distinguishing between hot and cold water” (*p* value < 0.05). Except for “difficulty distinguishing hot/cold water” (*p* value 0.128) and “cramps in feet” (*p* value > 0.999), also TIPN symptoms rated as “a little” had a significant negative effect on GHS/QoL when compared with unaffected survivors, data not shown.

### Prevalence of clinically important impairment of functioning and personal finances

**Fig. 3:**
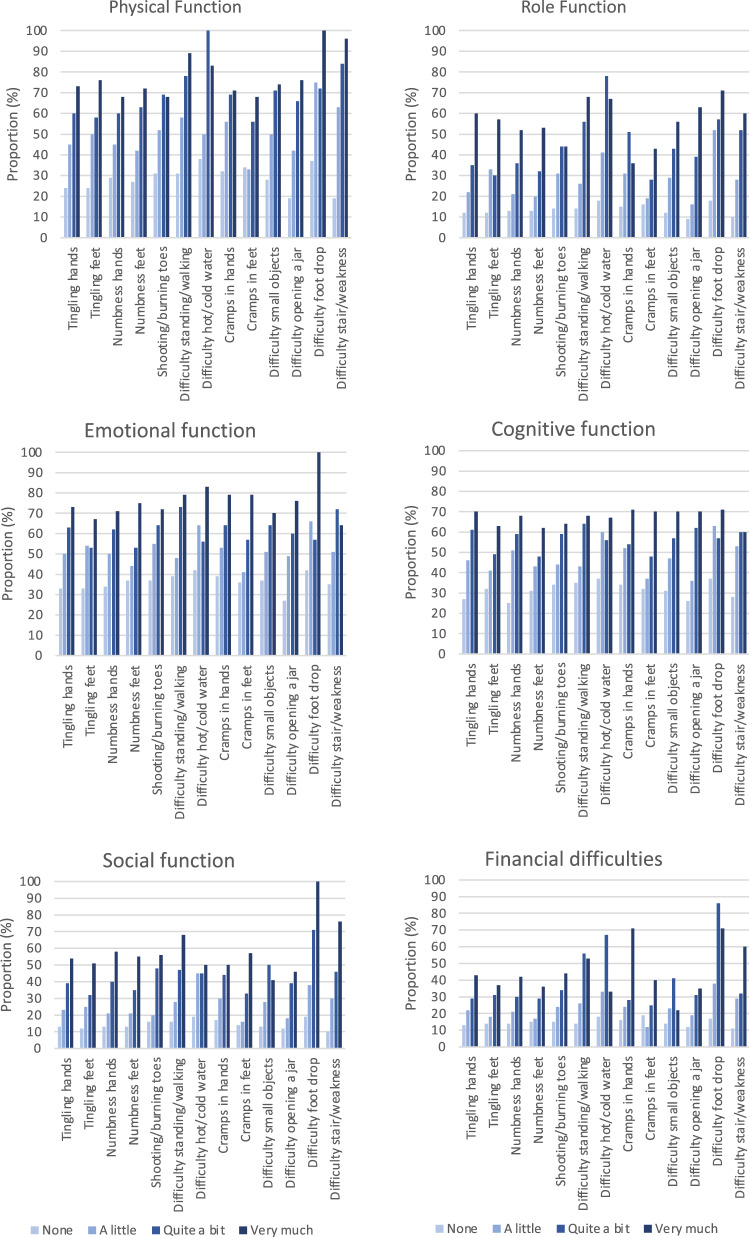
Prevalence rate of survivors with functional health and financial difficulties of clinical importance (threshold of clinical importance), classified by reported level of 13 peripheral neuropathy symptoms from the EORTC-CIPN20. The bars represent “Not at all,” “A little,” “Quite a bit,” or “Very much” of the symptoms

The prevalence rate (or proportion) of ESBC survivors with moderate-severe TIPN symptoms who had mean scores of functional scales less than or mean scores of FI scale higher than the threshold values reported by Giesinger et al. varied between 29.5% and 93.3%. (Table 2). More than every second survivor with moderate-severe “difficulty walking because of foot drop” (64.3–85.7%) and “problems standing or walking because of difficulty feeling ground under feet” (53.1–81.3%) reported an impairment of all functional scales and personal finances. Also, TIPN symptoms rated as “a little” was associated with a detrimental effect on functioning and FI (Supplementary Table 1). The proportion of affected survivors varied between 16.0% (RF, “difficulty opening a jar”) and 75.0% (PF, “difficulty walking because of foot drop”). The impact of increased severity of TIPN on functioning and personal finances is illustrated in (Fig. 3).

**Table 2:** The prevalence rate (proportion) of early-stage breast cancer survivors with moderate-severe symptoms of persistent peripheral neuropathy whose impact on self-perceived functional health and financial difficulties was of clinical importance

	PF *n*/*N* (%)	RF *n*/*N* (%)	EF *n*/*N* (%)	CF *n*/*N* (%)	SF *n*/*N* (%)	FI *n*/*N* (%)
Tingling fingers/hands	77/120 (64.2)	51/121 (42.1)	79/120 (65.8)	77/121 (63.6)	53/121 (43.8)	40/121 (33.1)
Tingling toes/feet	95/148 (64.2)	59/149 (39.6)	86/149 (57.7)	80/149 (53.7)	57/149 (38.2)	49/149 (32.9)
Numbness fingers/hands	74/120 (61.7)	48/121 (39.7)	78/121 (64.5)	74/121 (61.2)	54/121 (44.6)	40/121 (33.1)
Numbness toes/feet	99/151 (65.6)	59/152 (38.8)	91/152 (59.9)	79/152 (52.0)	63/152 (41.4)	47/152 (30.9)
Shooting/burning in feet	61/89 (68.5)	39/89 (43.8)	59/89 (66.3)	54/89 (60.7)	45/89 (50.6)	33/89 (37.1)
Problems standing/walking because difficulty feeling ground under feet	52/64 (81.3)	38/64 (59.4)	48/64 (75.0)	42/64 (65.6)	34/64 (53.1)	35/64 (54.7)
Difficulty distinguishing between hot/cold water	14/15 (93.3)	11/15 (73.3)	10/15 (66.7)	9/15 (60.0)	7/15 (46.7)	8/15 (53.3)
Cramps in hands	37/53 (69.8)	25/53 (47.2)	36/53 (67.9)	31/53 (58.5)	24/53 (45.3)	21/53 (39.6)
Cramps in feet	98/165 (59.4)	53/166 (31.9)	105/166 (63.3)	90/166 (54.2)	66/166 (39.8)	49/166 (29.5)
Difficulty manipulating small objects with fingers	60/83 (72.3)	39/83 (47.0)	55/83 (66.3)	51/83 (61.4)	39/83 (47.0)	29/83 (34.9)
Difficulty opening a jar or bottle because of weakness in hands	106/154 (68.8)	72/155 (46.5)	100/155 (64.5)	100/155 (64.5)	64/155 (41.3)	50/155 (40.0)
Difficulty walking because of foot drop	12/14 (85.7)	9/14 (64.3)	11/14 (78.6)	9/14 (64.3)	12/14 (85.7)	11/14 (78.6)
Difficulty climbing stairs or getting up/out of chair because of weakness in legs	80/92 (87.0)	50/93 (53.8)	65/93 (69.9)	56/93 (60.2)	50/93 (36.2)	37/93 (39.8)

In accordance with Giesinger et al. 2020[12]

*PF* physical functioning, *RF* role functioning, *EF* emotional functioning, *CF* cognitive functioning,

*SF* social functioning, *FI* financial difficulties

### Magnitude of clinical relevance

The CIDs, as defined by Cock et al. [11], between survivors with and without TIPN was most profound for “numbness in toes and feet” and “difficulty walking because of foot drop,” see Supplementary Table 2. Both symptoms had a clinically relevant impact (medium CID) on GHS/QoL. For ESBCS with moderate-severe symptoms of TIPN, “difficulty climbing stairs or getting out of chair because of weakness in legs” and “problems standing or walking because of difficulty feeling ground under feet” were associated with the largest CIDs (medium to large) on all scales (Table 3). The estimated largest CID between survivors with and without TIPN was found for social functioning (Supplementary Table 3).

**Table 3:**
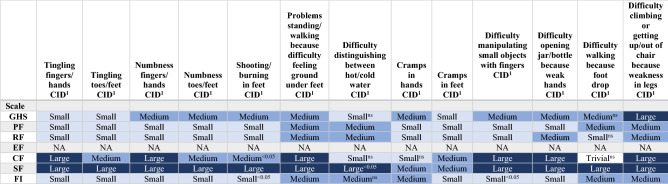
The estimated magnitude of clinically important difference of adjusted mean scores between early-stage breast cancer survivors without or a little compared with those with moderate-severe persistent taxane-induced peripheral neuropathy on GHS/QoL, functional health, and personal finances

The estimated differences in adjusted mean scores between survivors with and without persistent TIPN were all significant except for the symptom “difficulty distinguishing between hot/cold water” (ns for GHS, EF, CF, FI) (Supplementary table 4)

The estimated differences in adjusted mean scores between survivors without to “a little” versus those with moderate-severe persistent TIPN were all significant except for the symptoms “cramps in hands” (ns for CF), “difficulty distinguishing between hot/cold water” (ns for GHS, EF, CF, FI) and “difficulty walking because of foot drop” (ns for GHS, RF, EF, and CF) (Supplementary table 4)

*CID* clinical important difference, *GHS* Global Health Status/quality of life, *PF* physical functioning, *RF* role functioning, *EF* emotional functioning, *CF* cognitive functioning, *SF* social functioning, *FI* financial difficulties due to the problem, *NA* not applicable, *NS* not significant

^1^The guidelines by Cocks et al. 2011 [11] were used to interpret the difference in adjusted mean scores (Supplementary Table 3). CID was categorized into four groups: The mean difference in scores was categorized into four groups depending on their estimated clinical relevance: a large difference was defined as one representing unequivocal clinical relevance; a median difference as clinically relevant but to a lesser extent; a small difference as clinically relevant but subtle; and a trivial difference as circumstances unlikely to have any clinical relevance. The emotional functioning subscale was omitted in the guidelines. The Bonferroni method was used to correct for multiple comparisons. The differences in adjusted mean scores all have *p* values < 0.01, except when marked < 0.05 or ns in the table

The unadjusted and adjusted mean scores of GHS/QoL, functional scales, and FI among ESBC survivors are shown in Supplementary Table 3 and 4. The estimated difference in adjusted mean score between survivors with and those without persistent TIPN were all significant, except for “difficulty distinguishing between hot/cold water” (not significant, ns, for GHS, EF, CF, and FI), “cramps in feet” (FI), and “difficulty walking because of foot drop” (EF, CF).

The estimated difference in adjusted mean score between survivors with moderate-severe TIPN and those without or “a little” TIPN, were all significant except for “cramps in hands” (CF), “difficulty distinguishing between hot/cold water” (ns for GHS, EF, CF, and FI), and “difficulty walking because of foot drop” (ns for GHS, RF, EF, and CF) (Supplementary Table 4). The number of missing responses for each scale and symptom was < 1% (data not shown).

### Consequences due to TIPN in hands and/or feet

**Fig. 4:**
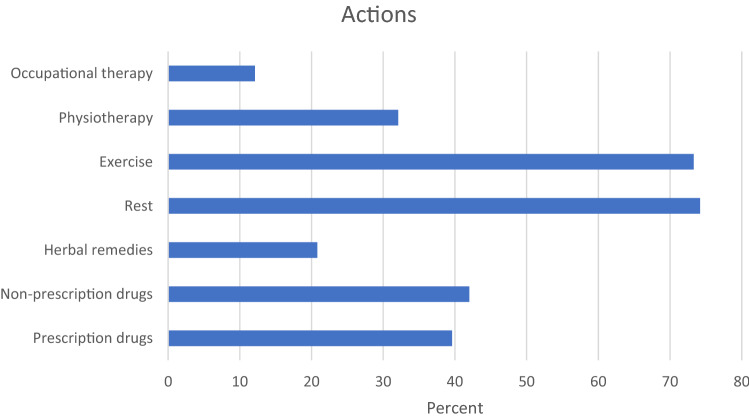
One or several actions taken by 239 survivors with persistent peripheral neuropathy in hands/feet to relieve their discomfort

Among survivors with TIPN symptoms in hands/feet, 69 out of 400 (17.3%) reported they had used one or several aids (e.g., wheelchair, walker, crutches) during the previous six months due to their PN. Also, other actions were taken to relief their discomfort. The most common actions were rest (74.5%) and exercise (73.6%), followed by non-prescription drugs (42.3%), prescription drugs (39.7%), physiotherapy (32.2%), herbal remedies (20.9%), and occupational therapy (12.1%), see Fig. 4. Half of affected survivors had discussed their problem with a physician or nurse, whereas 15.3% had not spoken to anyone. Among the 379 women younger than the normal retirement age of 65 years, 4.7% reported they had been on sick leave due to TIPN in hands or feet for more than three months during the previous six months and 2.1% reported a disability pension due to TIPN (data not shown).

Among survivors with TIPN in hands/feet 41.8% reported that the symptoms would bother them moderately to a lot if they persisted lifelong. Survivors reporting moderate-severe “tingling in toes/feet,” “numbness in toes/feet,” and/or “cramps in feet,” 7%, 8%, and 7% agreed “quite a bit” or “very much” with the statement *“If I had known about the long-term effects of the chemotherapy, I would have abstained from the treatment, even if it had shortened my life”* compared to 3% among those reporting none or a little of those symptoms (*p* < 0.05, data not shown).

## Discussion

We previously reported an excess risk for 13 individual symptoms of persistent sensory and motor peripheral neuropathy among recurrence-free ESBC survivors treated up to 7 years earlier with taxane chemotherapy regimens versus women without prior cancer. In the present study, we found that survivors with persistent TIPN symptoms had worse global QoL, functioning, and personal finances compared with unaffected survivors. The clinical important impairment of HRQL was most profound for “numbness in toes/feet” and “difficulty walking because of foot drop.” The prevalence rate of survivors with moderate-severe TIPN symptoms who had impaired function and financial difficulties varied but exceeded every second survivor with “difficulty walking because of foot drop” and “problems standing/walking because of difficulty feeling ground under feet.” The impairment of HRQL worsened with increased severity of TIPN symptoms but also TIPN symptoms rated as “a little” were associated with a detrimental effect on HRQL.

To the best of our knowledge, only two previous studies have presented data on the impact of persistent TIPN on HRQL among ESBCS. In a study by Eckhoff et al., 1031 women post-docetaxel treatment of ESBC self-reported on PN using a non-validated questionnaire corresponding to the clinician-reported National Cancer institute Common Terminology Criteria of Adverse Events (NCI-CTCAE version 2.0) and the EORTC QLQ-C30 was used to assess HRQL 1–3 years after diagnosis [17]. The results showed that sensory PN grade 2 was associated with medium CID for global QoL and grade 3–4 with large CID. The study by Bandos et al. reporting on the impact of tingling/numbness in hands or feet on QoL, using the FACT-B-TOI in ESBCS 2 years after docetaxel treatment [6], also showed that increased severity of sensory TIPN symptoms significantly worsened QoL. These findings are in accordance with our results but notably the median time since treatment in our study was longer (3.6 years; 1.5–7.3 years) which emphasizes the seriousness and importance of persistent TIPN. In contrast to both previous studies, we report on the impact of specific sensory and motor symptoms on HRQL. Our results indicate that the impact on HRQL varies with both the specific symptom as well as the severity of the TIPN. In addition, we have not found any other study that also reports on TIPN rated as mild, i.e., “a little”, concerning HRQL.

Moderate-severe numbness and tingling in toes/feet, two common persistent sensory TIPN symptoms affecting 23% and 24% of our study population, respectively [13], had a clinically important impact on global QoL. In addition, over 60% of these survivors reported deterioration of physical function, indicating that at least every seventh ESBCS exposed to (neo)adjuvant taxane treatment during primary therapy will suffer from moderate-severe numbness/tingling in toes/feet for many years, perhaps lifelong, after treatment. More than every second ESBCS with moderate-severe “difficulty walking because of foot drop” and “problems standing/walking because of difficulty feeling ground under feet” reported an impairment of all functional scales, 79% and 55%, respectively, also reported financial difficulties of clinical importance. Although these two TIPN symptoms are relatively rare, 2% and 10%, respectively [13], the consequences of these side effects for those affected are devastating and every effort should be taken to prevent their occurrence. In addition to moderate-severe “problems standing/walking because of difficulty feeling ground under feet”, also “difficulty climbing stairs or getting out of chair because of weakness of legs” was associated with the largest CIDs (medium to large) on all scales. The latter symptom occurred in 14% of ESBCS [13]. We can only speculate on how TIPN may impact personal finances. Sensory or motor TIPN symptoms may hinder the possibility to pursue a profession as indicated by ESBCS reporting sick leave or disability pension due to TIPN several years after treatment.

The clinical implication of our findings is that caregivers should not only monitor sensory (e.g., difficulty feeling ground under feet) and motor symptoms (weakness of legs) but also inquire if, how, and to what extent daily life functioning is limited because of the symptom.

Our results suggest that persistent PN is a common side effect after (neo)adjuvant taxane treatment which clearly affect HRQL among ESBCS. Taxane-containing adjuvant chemotherapy regimens have been shown to improve overall survival and disease-free survival compared with non-taxane-containing regimens in women with ESBC [18, 19]. In the recent Cochrane systematic review, the authors also concluded that taxanes likely lead to a large risk of severe neuropathy, but based on seven of the 29 studies included in the review, the results indicated that taxanes may make little or no difference in QoL compared with chemotherapy without a taxane. However, the evidence was considered of low quality. We did not include a control group of ESBCS previously treated with non-taxane-containing adjuvant chemotherapy. Instead, we explored the long-term impact of persistent individual TIPN symptoms on HRQL in a population-based cohort of ESBCS and compared the results with unaffected survivors. The results of real-world population-based data are important complement to randomized trials.

Considering that most adjuvant ESBC patients have an excellent prognosis, the risk of persistent TIPN and its consequences should be considered in treatment planning. For instance, in an older woman with diabetes mellitus and overweight, the risk of deteriorating HRQL could contribute to a decision, together with the patient, to abstain from a taxane in an adjuvant chemotherapy regimen. As shown in our study, some ESBCSs affected by PN stated that they would have abstained from treatment if they had known the consequences, indicating that careful communication about benefits and risks is needed with every patient.

CIPN is acknowledged as a serious clinical problem wherefore international clinical practice guidelines on the prevention and treatment of CIPN have been developed [20–22]. There is however an overall lack of evidence supporting the use of preventive approaches for CIPN although cryotherapy seems promising [23]. Also, the evidence for efficacious treatment of established CIPN is limited, except for neuropathic pain [24]. In our study, most survivors found exercise, and rest, to relieve their symptoms, the evidence that exercise and functional training reduce CIPN symptoms is growing [25]. Despite the current lack of strong evidence-based interventions to treat established CIPN it is essential to diagnose CIPN as early as possible to relieve the symptoms. It is therefore alarming that only every second ESBC survivors in our study had spoken to a nurse or doctor about their TIPN symptoms; hence, PN symptoms need more attention from health care providers. We therefore suggest that TIPN must be addressed specifically in international clinical guidelines of primary breast cancer so consideration will be made before final treatment decision.

Some of the strengths of our study are the large population-based cohort and the high response rate. Validated instruments and privately answered questionnaire lower the risk of bias. The number of missing data was very low. We included only recurrence-free women; therefore symptoms, psychological impact, or treatment effects related to residual disease could not affect our results. We adjusted mean scores for possible confounders regarding HRQL evaluations and corrected for multiple comparisons. However, we had no data on pre-treatment co-morbidities, PN, or HRQL due to the cross-sectional study design. We cannot exclude the possibility that non-participants would have answered differently.

In conclusion, persistent sensory and motor TIPN symptoms among long-term ESBC survivors were associated with a clinically relevant detrimental effect on global QoL, functional health, and personal finances. The effect varied with specific symptom and the severity of TIPN but also TIPN symptoms rated as mild or “a little” should be noticed and monitored carefully. In clinical practice, the risk of persistent TIPN must be balanced with the estimated risk of recurrence and communicated with the individual patient. We therefore urge international clinical guidelines to specifically address the risk of TIPN. Further research on prevention and risk factors including genetic variants is warranted, as is research on how to relieve symptoms and improve HRQL among affected ESBC survivors.

## Supplementary Information

Below is the link to the electronic supplementary material.

Supplementary file1 (DOCX 96 kb)

Supplementary file2 (DOCX 22 kb)

## Data Availability

The dataset analyzed during the current study is available from the corresponding author on reasonable request.
