# Supplementary material for: Impact of persistent peripheral neuropathy on health-related quality of life among early-stage breast cancer survivors: a population-based cross-sectional study
Source: Breast Cancer Res Treat. 2022 Aug 9;195(3):379–91. doi: 10.1007/s10549-022-06670-9 (PMC9464756; doi:10.1007/s10549-022-06670-9)
Supplement: Supplementary file 1 — Supplementary file1 (DOCX 96 kb) [file 10549_2022_6670_MOESM1_ESM.docx]

**Supplementary Table 1.** The prevalence rates of early-stage breast cancer survivors with sensory and motor persistent peripheral neuropathy

symptoms reported as “a little” whose impact on self-perceived functional health and financial difficulties was of clinical importance^1^.

|  | **PF**  **n/N (%)** | **RF**  **n/N (%)** | **EF**  **n/N (%)** | **CF**  **n/N (%)** | **SF**  **n/N (%)** | **FI**  **n/N (%)** |
| --- | --- | --- | --- | --- | --- | --- |
| **Tingling fingers/hands** | 85/190  (44.7%) | 42/189  (22.2%) | 94/190  (49.5%) | 88/190  (46.3%) | 43/190  (22.6%) | 42/190  (22.1%) |
| **Tingling toes/feet** | 79/159  (49.7%) | 33/158  (20.9%) | 85/159  (53.5%) | 65/159  (40.9%) | 40/159  (25.2%) | 29/159  (18.2%) |
| **Numbness fingers/hands** | 84/188  (44.7%) | 40/188  (21.3%) | 93/188  (49.5%) | 95/188  (50.5%) | 40/188 (21.3%) | 40/188 (21.3%) |
| **Numbness toes/feet** | 65/156  (41.7%) | 31/156  (19.9%) | 69/156  (44.2%) | 67/156  (42.9%) | 33/156  (21.2%) | 27/156  (17.3%) |
| **Shooting/burning in feet** | 53/102  (52.0%) | 32/102  (31.4%) | 56/102  (54.9%) | 45/102  (44.1%) | 20/102  (19.6%) | 24/102  (23.5%) |
| **Problems standing/walking because difficulty feeling ground under feet** | 54/93  (58.1%) | 24/92  (26.1%) | 45/93  (48.4%) | 40/93  (43.0%) | 26/93  (28.0%) | 24/93  (25.8%) |
| **Difficulty distinguishing between hot/cold water** | 21/42  (50.0%) | 17/42  (40.5%) | 27/42  (64.3%) | 25/42  (59.5%) | 19/42  (45.2%) | 14/42  (33.3%) |
| **Cramps in hands** | 67/119  (56.3%) | 37/120  (30.8%) | 64/120  (53.3%) | 62/120  (51.7%) | 36/120  (30.0%) | 29/120  (24.2%) |
| **Cramps in feet** | 64/195  (32.8%) | 36/195  (18.5%) | 79/195  (40.5%) | 73/195  (37.4%) | 32/195  (16.4%) | 30/119  (25.2%) |
| **Difficulty manipulating small objects with fingers** | 88/175  (50.3%) | 50/175  (28.6%) | 90/176  (51.1%) | 82/176  (46.6%) | 50/176  (28.4%) | 40/176  (22.7%) |
| **Difficulty opening a jar or bottle because of weakness in hands** | 101/239  (42.3%) | 38/238  (16.0%) | 116/238  (48.7%) | 86/239  (36.0%) | 44/239  (18.4%) | 45/239  (18.8%) |
| **Difficulty walking because of foot drop** | 24/32  (75.0%) | 16/31  (51.6%) | 21/32  (65.6%) | 20/32  (62.5%) | 12/32  (37.5%) | 12/32  (37.5%) |
| **Difficulty climbing stairs or getting up/out of chair because of weakness in legs** | 102/161  (63.4%) | 45/160  (28.1%) | 83/161  (51.6%) | 86/161  (53.5%) | 48/161  (29.8%) | 46/161  (28.6%) |

Abbreviations: PF, physical functioning; RF, role functioning; EF, emotional functioning; CF, cognitive functioning, SF,

social functioning; FI, Financial difficulties.

^1^ In accordance with Giesinger et al 2020 [12]

**Supplementary Table 2.** The estimated magnitude of clinically important difference of adjusted mean scores between early-stage breast cancer survivors

without and with any severity (a little, quite a bit, very much) of persistent taxane-induced peripheral neuropathy on global health status/quality of life, functional health, and personal finances.

|  | Tingling  fingers/  hands  CID^1^ | Tingling  toes/feet  CID^1^ | Numbness  fingers/  hands  CID^1^ | Numbness  toes/feet CID^1^ | Shooting/  burning  in feet  CID^1^ | Problems standing/  walking because difficulty feeling ground under feet  CID^1^ | Difficulty distinguishing between hot/cold water  CID^1^ | Cramps in hands  CID^1^ | Cramps in feet  CID^1^ | Difficulty  manipulating  small objects with fingers  CID^1^ | Difficulty  opening  jar/bottle  because  weak  hands  CID^1^ | Difficulty  walking  because  foot  drop  CID^1^ | Difficulty  climbing or getting  up/out of chair  because weakness in legs  CID^1^ |
| --- | --- | --- | --- | --- | --- | --- | --- | --- | --- | --- | --- | --- | --- |
| Scale |  |  |  |  |  |  |  |  |  |  |  |  |  |
| GHS | Small | Small | Small | Medium | Small | Small | Trivial^ns^ | Small | Small | Small | Small | Medium | Small |
| PF | Small | Small | Small | Small | Small | Medium | Small | Small | Trivial | Small | Small | Medium | Small |
| RF | Small | Small | Small | Small | Small | Small | Small | Small | Small | Small | Small | Small | Small |
| EF | NA | NA | NA | NA | NA | NA | NA | NA | NA | NA | NA | NA | NA |
| CF | Medium | Medium | Small | Medium | Small | Small | Small^ns^ | Medium | Small | Medium | Medium | Small^ns^ | Medium |
| SF | Medium | Large | Medium | Medium | Medium | Large | Large | Medium | Small | Large | Medium | Large | Large |
| FI | Small | Small | Small | Small | Small^<0.05^ | Small | Small^ns^ | Small | Trivial^ns.^ | Small | Small^<0.05^ | Medium | Small |

Abbreviations: CID, clinical important difference; GHS, Global Health Status/quality of life; PF, physical functioning; RF, role functioning; EF, emotional functioning; CF, cognitive functioning, SF, social functioning; FI, financial difficulties due to the problem; NA, not applicable; ns, not significant.

^1^ The guidelines by Cocks et al 2011 [11] were used to interpret the difference in adjusted mean scores (Supplementary Table 3). CID was categorized into four groups: The mean difference in scores was categorized into four groups depending on their estimated clinical relevance: a large difference was defined as one representing unequivocal clinical relevance; a median difference as clinically relevant but to a lesser extent; a small difference as clinically relevant but subtle; and a trivial difference as circumstances unlikely to have any clinical relevance. The emotional functioning subscale was omitted in the guidelines. The Bonferroni method was used to correct for multiple comparisons. The differences in adjusted mean scores all have p-values <0.01, except when marked <0.05 or ns in the table.

Footnote: The estimated differences in adjusted mean scores between survivors with and without persistent TIPN were all significant except for the symptom “difficulty distinguishing between hot/cold water” (ns for GHS, EF, CF, FI), “cramps in feet” (FI) and “difficulty walking because of foot drop” (EF, CF) (Supplementary Table 3).

**Supplementary Table 3.** The impact of 13 individual symptoms of sensory and motor taxane-induced peripheral neuropathy on global health status/quality of life, functional health, and finances in early-stage breast cancer survivors. Unadjusted and adjusted mean (SE) scores of each scale (EORTC QLQ-C30 instrument) and the difference of the adjusted mean scores were calculated.

|  | Tingling fingers/hands | | | Tingling toes/feet | | | Numbness fingers/hands | | | Numbness toes/feet | | |
| --- | --- | --- | --- | --- | --- | --- | --- | --- | --- | --- | --- | --- |
| Scale | **Not at all**  Unadjusted^1^  Adjusted^2^  Mean (SE) | **Any severity**  Unadjusted^1^  Adjusted^2^  Mean (SE) | Δ^3^  ClD^4^ | **Not at all**  Unadjusted^1^  Adjusted^2^  Mean (SE) | **Any severity**  Unadjusted^1^  Adjusted^2^  Mean (SE) | Δ^3^  ClD^4^ | **Not at all**  Unadjusted^1^  Adjusted^2^  Mean (SE) | **Any severity**  Unadjusted^1^  Adjusted^2^  Mean (SE) | Δ^3^  ClD^4^ | **Not at all**  Unadjusted^1^  Adjusted^2^  Mean (SE) | **Any severity**  Unadjusted^1^  Adjusted^2^  Mean (SE) | Δ^3^  ClD^4^ |
| GHS, no | 331  73.6 (1.1)  61.3 (2.7) | 310  63.5 (1.2) ^**^  54.2 (2.6) ^**^ | -7.1  Small | 334  74.7 (1.1)  63.5 (2.7) | 307  62.5 (1.2) ^**^  53.7 (2.5) ^**^ | -9.8  Small | 331  74.0 (1.1)  61.7 (2.7) | 308  63.2 (1.2) ^**^  54.3 (2.6) ^**^ | -7.4  Small | 333  74.6 (1.1)  63.8 (2.7) | 307  62.6 (1.2) ^**^  53.6 (2.5) ^**^ | -10.2  Medium |
| PF, no | 331  86.8 (0.9)  76.9 (2.1) | 310  77.7 (1.1)^**^  70.0 (2.0) ^**^ | -6.9  Small | 334  87.7 (0.9)  77.5 (2.1) | 307  76.7 (1.1) ^**^  70.2 (2.0) ^**^ | -7.3  Small | 331  87.0 (0.9)  77.0 (2.1) | 308  77.5 (1.1) ^**^  70.0 (2.0) ^**^ | -7.0  Small | 333  87.6 (0.9)  78.2 (2.1) | 307  76.8 (1.2) ^**^  69.8 (2.0) ^**^ | -8.4  Small |
| RF, no | 329  84.6 (1.3)  73.6 (3.3) | 310  68.4 (1.6) ^**^  60.5 (3.1) ^**^ | -13.1  Small | 332  84.3 (1.3)  73.6 (3.3) | 307  68.6 (1.6) ^**^  61.4 (3.1) ^**^ | -12.2  Small | 328  83.6 (1.4)  72.2 (3.3) | 309  69.5 (1.5) ^**^  61.6 (3.2) ^**^ | -10.6  Small | 330  83.5 (1.3)  73.3 (3.3) | 308  69.6 (1.6) ^**^  61.6 (3.1) ^**^ | -11.7  Small |
| EF, no | 330  77.7 (1.2)  70.0 (3.1) | 310  64.6 (1.5) ^**^  59.3 (3.0) ^**^ | -10.7  NA^4^ | 332  76.9 (1.2)  71.0 (3.1) | 308  65.6 (1.5) ^**^  59.5 (3.0) ^**^ | -11.5  NA^4^ | 330  77.7 (1.2)  70.0 (3.1) | 309  64.8 (1.5) ^**^  59.4 (3.0) ^**^ | -10.6  NA^4^ | 331  76.0 (1.2)  70.0 (3.1) | 308  66.6 (1.5) ^**^  60.2 (3.0) ^**^ | -9.8  NA^4^ |
| CF, no | 330  81.9 (1.2)  71.7 (3.2) | 311  67.6 (1.6) ^**^  60.7 (3.0) ^**^ | -11.0  Medium | 333  79.9 (1.2)  71.4 (3.2) | 308  69.8 (1.6) ^**^  61.6 (3.0) ^**^ | -9.8  Medium | 330  82.6 (1.2)  73.0 (3.1) | 309  66.9 (1.5) ^**^  60.0 (3.0) ^**^ | -7.0  Small | 332  79.6 (1.3)  71.7 (3.2) | 308  70.1 (1.6) ^**^  61.5 (3.0) ^**^ | -10.2  Medium |
| SF, no | 330  83.2 (1.2)  71.7 (3.2) | 311  67.9 (1.6) ^**^  60.7 (3.0) ^**^ | -11.0  Medium | 333  83.9 (1.2)  78.3 (3.3) | 308  67.1 (1.6) ^**^  63.0 (3.2) ^**^ | -15.3  Large | 330  82.9 (1.3)  75.8 (3.4) | 309  68.0 (1.6) ^**^  63.7 (3.3) ^**^ | -12.1  Medium | 332  83.2 (1.2)  78.1 (3.3) | 308  67.6 (1.6) ^**^  63.3 (3.2) ^**^ | -14.8  Medium |
| FI,  no | 330  6.2 (1.0)  22.2 (2.8) | 311  14.9 (1.6) ^**^  28.4 (2.7) ^**^ | +6.2  Small | 333  6.8 (1.0)  22.1 (2.8) | 308  14.3 (1.6) ^**^  28.2 (2.7) ^**^ | +6.1  Small | 330  6.5 (1.0)  22.3 (2.8) | 309  14.7 (1.6) ^**^  28.4 (2.7) ^**^ | +6.1  Small | 332  7.4 (1.1)  22.1 (2.8) | 308  13.6 (1.6) ^**^  28.1 (2.7) ^**^ | +6.0  Small |

|  | Shooting/burning in feet | | | Problems standing/walking because of difficulty feeling ground under feet | | | Difficulty distinguishing between hot/cold water | | |
| --- | --- | --- | --- | --- | --- | --- | --- | --- | --- |
| Scale | **Not at all**  Unadjusted^1^  Adjusted^2^  Mean (SE) | **Any severity**  Unadjusted^1^  Adjusted^2^  Mean (SE) | Δ^3^  ClD^4^ | **Not at all**  Unadjusted^1^  Adjusted^2^  Mean (SE) | **Any severity**  Unadjusted^1^  Adjusted^2^  Mean (SE) | Δ^3^  ClD^4^ | **Not at all**  Unadjusted^1^  Adjusted^2^  Mean (SE) | **Any severity**  Unadjusted^1^  Adjusted^2^  Mean (SE) | Δ^3^  ClD^4^ |
| GHS, no | 449  72.6 (1.0)  61.5 (2.6) | 191  59.6 (1.6) ^**^  51.6 (2.7) ^**^ | -9.9  Small | 480  72.1 (0.9)  61.2 (2.6) | 157  58.7 (1.6) ^**^  51.9 (2.7) ^**^ | -9.3  Small | 582  69.9 (0.9)  58.1 (2.6) | 57  59.7 (2.8) ^**^  54.6 (3.4)^ns^ | -3.5  Trivial |
| PF, no | 449  86.2 (0.8)  76.7 (2.0) | 191  73.6 (1.5) ^**^  67.6 (2.1) ^**^ | -9.1  Small | 480  86.4 (1.7)  77.6 (2.0) | 157  70.4 (1.7) ^**^  66.3 (2.0) ^**^ | -11.3  Medium | 582  83.9 (0.7)  75.3 (2.0) | 57  67.7 (3.4) ^**^  64.2 (2.6) ^**^ | -11.1  Small |
| RF, no | 447  82.4 (1.1)  72.2 (3.2) | 191  63.8 (2.1) ^**^  57.4 (3.3) ^**^ | -14,8  Small | 479  82.1 (1.1)  72.5 (3.1) | 156  60.9 (2.3) ^**^  56.6 (3.3) ^**^ | -13.6  Small | 580  78.4 (1.1)  68.5 (3.2) | 57  58.8 (4.3) ^**^  56.6 (4.2) ^**^ | -11.9  Small |
| EF, no | 448  74.9 (1.1)  67.8 (3.1) | 191  63.2 (1.9) ^**^  58.3 (3.2) ^**^ | -9.5  NA^4^ | 480  74.1 (1.1)  67.3 (3.1) | 157  63.5 (2.1) ^**^  58.9 (3.2) ^**^ | -8.4  NA^4^ | 581  72.2 (1.0)  64.9 (3.1) | 57  63.6 (3.4) ^ns^  60.1 (4.0) ^ns^ | -4.8  NA^4^ |
| CF, no | 449  78.4 (1.1)  69.0 (3.1) | 191  66.8 (2.1) ^**^  60.1 (3.2) ^**^ | -8.9  Small | 480  77.7 (1.1)  69.0 (3.1) | 157  66.2 (2.3) ^**^  60.1 (3.3) ^**^ | -8.9  Small | 582  76.4 (1.0)  67.0 (3.1) | 57  61.4 (3.8) ^**^  59.1 (4.1) ^ns^ | -7.9  Small |
| SF, no | 449  80.7 (1.1)  74.3 (3.3) | 191  64.1 (2.1) ^**^  61.0 (3.4) ^**^ | -13.3  Medium | 480  80.6 (1.1)  75.6 (3.3) | 157  60.9 (2.4) ^**^  59.1 (3.4) ^**^ | -16.5  Large | 582  77.9 (1.0)  72.7 (3.3) | 57  53.8 (3.9) ^**^  54.0 (4.3) ^**^ | -18.7  Large |
| FI, no | 449  7.7 (1.0)  23.4 (2.8) | 191  16.7 (2.2) ^**^  29.3 (2.9) ^*^ | +5.9  Small | 480  6.9 (0.9)  22.1 (2.7) | 157  21.0 (2.5) ^**^  31.2 (2.8) ^**^ | +9.1  Small | 582  9.2 (0.9)  24.4 (2.7) | 57  22.2 (4.4) ^**^  31.6 (3.6) ^ns^ | +7.2  Small |

|  | Cramps in hands | | | Cramps in feet | | | Difficulty manipulating small objects with fingers | | | Difficulty opening a jar or bottle because of weakness in hands | | |
| --- | --- | --- | --- | --- | --- | --- | --- | --- | --- | --- | --- | --- |
| Scale | **Not at all**  Unadjusted^1^  Adjusted^2^  Mean (SE) | **Any severity**  Unadjusted^1^  Adjusted^2^  Mean (SE) | Δ^3^  ClD^4^ | **Not at all**  Unadjusted^1^  Adjusted^2^  Mean (SE) | **Any severity**  Unadjusted^1^  Adjusted^2^  Mean (SE) | Δ^3^  ClD^4^ | **Not at all**  Unadjusted^1^  Adjusted^2^  Mean (SE) | **Any severity**  Unadjusted^1^  Adjusted^2^  Mean (SE) | Δ^3^  ClD^4^ | **Not at all**  Unadjusted^1^  Adjusted^2^  Mean (SE) | **Any severity**  Unadjusted^1^  Adjusted^2^  Mean (SE) | Δ^3^  ClD^4^ |
| GHS, no | 465  72.1 (0.9)  60.7 (2.6) | 172  60.0 (1.8) ^**^  52.8 (2.7) ^**^ | -7.9  Small | 281  72.4 (1.2)  61.1 (2.7) | 360  66.0 (1.2) ^**^  55.3 (2.6) ^**^ | -5.8  Small | 383  73.4 (1.0)  61.7 (2.7) | 258  61.8 (1.4) ^**^  53.8 (2.6) ^**^ | -7.9  Small | 249  76.4 (1.2)  64.1 (2.8) | 393  64.1 (1.1) ^**^  54.9 (2.5) ^**^ | -9.2  Small |
| PF, no | 465  86.0 (0.8)  76.1 (2.0) | 172  73.0 (1.7) ^**^  68.5 (2.1) ^**^ | -7.6  Small | 281  85.8 (1.0)  76.0 (2.1) | 360  79.7 (1.0) ^**^  71.2 (2.0) ^**^ | -4.8  Trivial | 383  87.8 (0.8)  77.6 (2.0) | 258  74.5 (1.3) ^**^  69.1 (2.0) ^**^ | -8.5  Small | 249  90.9 (0.8)  80.0 (2.1) | 393  77.2 (1.0) ^**^  70.3 (1.9) ^**^ | -9.7  Small |
| RF, no | 462  81.5 (1.1)  71.2 (3.2) | 173  64.6 (2.2) ^**^  58.9 (3.3) ^**^ | -12.3  Small | 278  81.1 (1.5)  70.0 (3.9) | 361  73.3 (1.4) ^**^  63.8 (3.2) ^*^ | -6.2  Small | 381  84.3 (1.2)  74.0 (3.2) | 258  65.6 (1.8) ^**^  59.4 (3.2) ^**^ | -14.6  Small | 247  87.8 (1.4)  76.3 (3.4) | 393  70.0 (1.4) ^**^  62.1 (3.1) ^**^ | -16.0  Small |
| EF, no | 464  75.0 (1.0)  68.4 (3.1) | 173  62.0 (2.1) ^**^  57.4 (3.2) ^**^ | -11.0  NA^4^ | 279  76.6 (1.3)  70.0 (3.2) | 361  67.4 (1.4) ^**^  60.5 (3.0) ^**^ | -9.5  NA^4^ | 381  75.6 (1.1)  68.6 (3.1) | 259  65.1 (1.6) ^**^  59.8 (3.1) ^**^ | -8.8  NA^4^ | 248  80.2 (1.3)  73.2 (3.2) | 393  66.0 (1.3) ^**^  60.3 (2.9) ^**^ | -12.9  NA^4^ |
| CF, no | 464  78.8 (1.1)  70.1 (3.1) | 173  65.0 (2.3) ^**^  58.4 (3.2) ^**^ | -11.6  Medium | 280  79.2 (1.4)  70.0 (3.2) | 361  71.7 (1.4) ^**^  62.8 (3.1) ^**^ | -7.2  Small | 382  80.3 (1.1)  71.8 (3.1) | 259  67.1 (1.8) ^**^  60.0 (3.1) ^**^ | -11.8  Medium | 248  83.1 (1.3)  73.2 (3.3) | 394  70.1 (1.4) ^**^  62.4 (3.0) ^**^ | -10.8  Medium |
| SF, no | 464  80.2 (1.1)  74.1 (3.3) | 173  64.0 (2.2) ^**^  61.2 (3.4) ^**^ | -12.9  Medium | 280  81.6 (1.4)  74.5 (3.5) | 361  71.5 (1.5) ^**^  65.5 (3.3) ^**^ | -9  Small | 382  82.7 (1.2)  77.1 (3.3) | 259  65.6 (1.8) ^**^  62.0 (3.2) ^**^ | -15.1  Large | 248  86.2 (1.4)  79.0 (3.5) | 394  70.0 (1.3) ^**^  65.0 (3.2) ^**^ | -14  Medium |
| FI, no | 464  7.6 (2.5)  23.2 (2.8) | 173  17.7 (2.5) ^**^  29.6 (2.9) ^**^ | +6.4  Small | 280  8.8 (1.3)  24.1 (2.9) | 361  11.6 (1.4) ^ns^  26.9 (2.8) ^ns^ | +2.8  Trivial | 382  7.0 (1.0)  22.1 (2.8) | 259  15.3 (1.8) ^**^  28.9 (2.8) ^**^ | +6.8  Small | 248  6.1 (1.2)  22.1 (3.0) | 394  13.1 (1.4) ^**^  27.3 (2.7) ^*^ | +5.2  Small |

|  | Difficulty walking  because of foot drop | | | | Difficulty climbing stairs or getting up/out of chair because of weakness in legs | | |
| --- | --- | --- | --- | --- | --- | --- | --- |
| Scale | **Not at all**  Unadjusted^1^  Adjusted^2^  Mean (SE) | **Any severity**  Unadjusted^1^  Adjusted^2^  Mean (SE) | | Δ^3^  ClD^4^ | **Not at all**  Unadjusted^1^  Adjusted^2^  Mean (SE) | **Any severity**  Unadjusted^1^  Adjusted^2^  Mean (SE) | Δ^3^  ClD^4^ |
| GHS, no | 592  70.4 (0.8)  58.7 (2.5) | 46  51.3 (3.2) ^**^  47.4 (3.7) ^**^ | | -11.3  Medium | 383  73.4 (1.0)  61.7 (2.7) | 258  61.8 (1.4) ^**^  53.8 (2.6) ^**^ | -7.9  Small |
| PF, no | 592  84.3 (0.7)  74.7 (1.9) | 46  61.3 (3.8) ^**^  60.4 (2.9) ^**^ | | -14.3  Medium | 383  87.8 (0.8)  77.6 (2.0) | 258  74.5 (1.3) ^**^  69.1 (2.0) ^**^ | -8.5  Small |
| RF, no | 591  78.9 (1.0)  68.0 (3.1) | | 45  50.0 (4.4) ^**^  50.1 (4.7) ^**^ | -17.9  Small | 381  84.3 (1.2)  74.0 (3.2) | 258  65.6 (1.8) ^**^  59.4 (3.2) ^**^ | -14.6  Small |
| EF, no | 592  72.5 (1.0)  64.8 (3.0) | | 46  57.6 (4.0) ^**^  56.1 (4.4) ^ns^ | -8.7  NA^4^ | 381  75.6 (1.1)  68.6 (3.1) | 259  65.1 (1.6) ^**^  59.8 (3.1) ^**^ | -8.8  NA^4^ |
| CF, no | 592  76.0 (1.0)  66.1 (3.0) | | 46  62.7 (4.3) ^*^  59.0 (4.5) ^ns^ | -7.1  Small | 382  80.3 (1.1)  71.8 (3.1) | 259  67.1 (1.8) ^**^  60.0 (3.1) ^**^ | -11.8  Medium |
| SF, no | 592  77.8 (1.0)  71.1 (3.2) | | 46  51.1 (4.1) ^**^  51.5 (4.7) ^**^ | -19.6  Large | 382  82.7 (1.2)  77.1 (3.3) | 259  65.6 (1.8) ^**^  62.0 (3.2) ^**^ | -15.1  Large |
| FI, no | 592  8.5 (0.9)  23.8 (2.6) | | 46  32.6 (5.7) ^**^  40.4 (3.9) ^**^ | +16.6  Medium | 382  7.0 (1.0)  22.1 (2.8) | 259  15.3 (1.8) ^**^  28.9 (2.8) ^**^ | +6.8  Small |

Abbreviations: CID, clinical important difference; GHS, Global Health Status; PF, physical functioning; RF, role functioning; EF, emotional functioning; CF, cognitive functioning, SF, social functioning; FI, financial difficulties due to the problem; NA, not applicable.

Higher scores on the QLQ-C30 global and functional scales indicate that the respondent has good general health and functional health. In contrast, a high score on FI, indicates worse financial difficulties due to the problem.

^1^Student´s t-test. ** = P <0.01, *= P <0.05, ns = non-significant. The Bonferroni method was used to adjust for multiple comparisons within each symptom.

^2^Linear regression (ANCOVA) adjusted for age, BMI, civil status, educational level, employment status, alcohol consumption, exercise, smoking, musculoskeletal disorders, cardiovascular disease, diabetes mellitus, pulmonary disease, and neurological disease. ** = P <0.01, *= P <0.05, ns = non-significant. The Bonferroni method was used to adjust for multiple comparisons within each symptom.

^3^ Δ = the difference of the adjusted mean scores between those with (a little/quite a bit/very much) and those without the symptom of peripheral neuropathy.

^4^ The guidelines by Cocks et al 2011 [11] were used to interpret the difference in mean scores. CID was categorized into four groups: a large difference was defined as one representing unequivocal clinical relevance; a median difference as clinically relevant but to a lesser extent; a small difference as clinically relevant but subtle; and a trivial difference as circumstances unlikely to have any clinical relevance. The emotional functioning subscale was omitted in the guidelines.

**Supplementary Table 4.** The impact of 13 individual symptoms of sensory and motor taxane-induced peripheral neuropathy, graded as moderate-severe, on global health status/quality of life, functional health, and finances in early-stage breast cancer survivors. Unadjusted and adjusted mean (SE) scores of each scale (EORTC QLQ C30 instrument) and the difference of the adjusted mean scores were calculated.

|  | Tingling fingers/hands | | | | Tingling toes/feet | | | | Numbness fingers/hands | | | Numbness toes/feet | | | |  |
| --- | --- | --- | --- | --- | --- | --- | --- | --- | --- | --- | --- | --- | --- | --- | --- | --- |
| Scale | | **Not at all-**  **A little**  Unadjusted^1^  Adjusted^2^  Mean (SE) | **Moderate-**  **Severe**  Unadjusted^1^  Adjusted^2^  Mean (SE) | Δ^3^  ClD^4^ | | **Not at all-**  **A little**  Unadjusted^1^  Adjusted^2^  Mean (SE) | **Moderate-**  **Severe**  Unadjusted^1^  Adjusted^2^  Mean (SE) | Δ^3^  ClD^4^ | **Not at all-**  **A little**  Unadjusted^1^  Adjusted^2^  Mean (SE) | **Moderate-**  **Severe**  Unadjusted^1^  Adjusted^2^  Mean (SE) | Δ^3^  ClD^4^ | | **Not at all-**  **A little**  Unadjusted^1^  Adjusted^2^  Mean (SE) | **Moderate-**  **Severe**  Unadjusted^1^  Adjusted^2^  Mean (SE) | Δ^3^  ClD^4^ | |
| GHS, n | | 521  71.3 (0.9)  59.9 (2.6) | 120  58.0 (1.9) ^**^  51.0 (2.9) ^**^ | -8.9  Small | | 493  71.9 (0.9)  61.1 (2.6) | 148  58.6 (1.8) ^**^  51.8 (2.7) ^**^ | -9.3  Small | 519  71.7 (0.9) 60.9 (2.6) | 120  56.4 (1.9) ^**^  49.8 (2.9) ^**^ | -11.1  Medium | | 489  72.4 (0.9)  62.4 (2.6) | 151  57.2 (1.8) ^**^  49.9 (2.7) ^**^ | -12.5  Medium | |
| PF, n | | 521  84.8 (0.8)  75.3 (2.0) | 120  71.9 (1.9) ^**^  67.1 (2.2) ^**^ | -8.2  Small | | 493  85.6 (0.8)  76.2 (2.0) | 148  72.0 (1.8) ^**^  70.0 (2.1) ^**^ | -6.2  Small | 519  85.1 (0.7)  76.0 (2.0) | 120  71.0 (2.1) ^**^  66.2 (2.2) ^**^ | -9.8  Small | | 489  86.2 (0.7)  77.4 (2.0) | 151  70.4 (1.8) ^**^  66.2 (2.1) ^**^ | -11.2  Small | |
| RF, n | | 518  80.7 (1.1)  71.2 (3.2) | 121  60.0 (2.6) ^**^  54.3 (3.5) ^**^ | -16.9  Small | | 490  81.4 (1.1)  72.3 (3.2) | 149  61.4 (2.3) ^**^  56.7 (3.3) ^**^ | -15.6  Small | 516  80.6 (1.1)  70.9 (3.2) | 121  60.5 (2.5) ^**^  55.4 (3.5) ^**^ | -15.5  Small | | 486  81.3 (1.1)  72.5 (3.2) | 152  62.4 (2.3) ^**^  56.4 (3.3) ^**^ | -16.1  Small | |
| EF, n | | 520  74.5 (1.0)  67.6 (3.0) | 120  58.0 (2.5) ^**^  55.0 (3.3) ^**^ | -12.6  NA^4^ | | 491  73.9 (1.0)  67.4 (3.1) | 149  63.5 (2.2) ^**^  58.6 (3.2) ^**^ | -9.0  NA^4^ | 518  74.7 (1.0)  68.2 (3.0) | 121  57.4 (2.6) ^**^  54.5 (3.3) ^**^ | -13.7  NA^4^ | | 487  74.1 (1.0)  68.7 (3.0) | 152  62.8 (2.2) ^**^  56.7 (3.2) ^**^ | -12.0  NA^4^ | |
| CF, n | | 520  78.6 (1.0)  69.9 (3.0) | 121  59.2 (2.7) ^**^  55.0 (3.4) ^**^ | -14.9  Large | | 492  77.9 (1.1)  69.7 (3.1) | 149  65.4 (2.4) ^**^  58.7 (3.3) ^**^ | -11.0  Medium | 518  78.8 (1.0)  70.4 (3.0) | 121  59.0 (2.8) ^**^  54.5 (3.4) ^**^ | -15.9  Large | | 488  77.6 (1.1)  70.1 (3.1) | 152  66.8 (2.4) ^**^  58.1 (3.2) ^**^ | -12.0  Medium | |
| SF, n | | 520  80.0 (1.1)  73.7 (3.3) | 121  59.4 (2.7) ^**^  57.6 (3.6) ^**^ | -16.1  Large | | 492  80.1 (1.1)  75.0 (3.3) | 149  61.8 (2.4) ^**^  59.5 (3.4) ^**^ | -15.5  Large | 518  80.0 (1.0)  74.5 (3.2) | 121  58.0 (2.8) ^**^  56.6 (3.6) ^**^ | -17.9  Large | | 488  80.5 (1.1)  76.7 (3.2) | 152  60.4 (2.4) ^**^  57.0 (3.4) ^**^ | -19.7  Large | |
| FI, n | | 520  8.1 (0.9)  23.6 (2.7) | 121  20.1 (3.0) ^**^  30.9 (3.0) ^**^ | +7.3  Small | | 492  7.7 (0.9)  22.9 (2.8) | 149  19.2 (2.6) ^**^  30.2 (2.9) ^**^ | +7.3  Small | 518  8.2 (0.9)  23.3 (2.7) | 121  21.1 (3.0) ^**^  31.4 (3.0) ^**^ | +8.1  Small | | 488  7.8 (0.9)  22.2 (2.7) | 152  18.6 (2.6) ^**^  31.3 (2.9) ^**^ | +9.1  Small | |

|  | Shooting/burning in feet | | | | Problems standing/ walking because difficulty feeling ground under feet | | | | Difficulty distinguishing between hot/cold water | | | |  |
| --- | --- | --- | --- | --- | --- | --- | --- | --- | --- | --- | --- | --- | --- |
| Scale | | **Not at all-**  **A little**  Unadjusted^1^  Adjusted^2^  Mean (SE) | **Moderate-**  **Severe**  Unadjusted^1^  Adjusted^2^  Mean (SE) | Δ^3^  ClD^4^ | | **Not at all-**  **A little**  Unadjusted^1^  Adjusted^2^  Mean (SE) | **Moderate-**  **Severe**  Unadjusted^1^  Adjusted^2^  Mean (SE) | Δ^3^  ClD^4^ | | **Not at all-**  **A little**  Unadjusted^1^  Adjusted^2^  Mean (SE) | **Moderate-**  **Severe**  Unadjusted^1^  Adjusted^2^  Mean (SE) | Δ^3^  ClD^4^ | |
| GHS, n | | 551  71.1 (0.9)  60.1 (2.6) | 89  54.2 (2.4) ^**^  48.7 (3.1) ^**^ | -11.4  Medium | | 573  70.6 (0.9)  59.4 (2.6) | 64  52.5 (2.5) ^**^  48.8 (3.4) ^**^ | -10.6  Medium | | 624  69.4 (0.8)  57.9 (2.6) | 15  48.9 (7.5) ^ns^  51.2 (6.2) ^ns^ | -6.7  Small | |
| PF, n | | 551  84.6 (0.7)  75.4 (2.0) | 89  69.0 (2.4) ^**^  65.0 (2.4) ^**^ | -10.4  Small | | 573  85.0 (0.7)  76.3 (1.9) | 64  60.3 (2.8) ^**^  59.0 (2.5) ^**^ | -17.3  Medium | | 624  83.3 (0.7)  74.6 (2.0) | 15  48.0(16.7) ^**^  52.9 (4.7) ^**^ | -21.7  Medium | |
| RF, n | | 549  80.0 (1.1)  69.6 (3.2) | 89  57.9 (2.9) ^**^  54.5 (3.8) ^**^ | -15.1  Small | | 571  80.0 (1.0)  70.3 (3.1) | 64  49.0 (3.4) ^**^  48.3 (4.0) ^**^ | -22.0  Medium | | 622  77.7 (1.0)  67.8 (3.2) | 15  35.6 (8.1) ^**^  43.7 (7.6) ^**^ | -24.1  Medium | |
| EF, n | | 550  73.8 (1.0)  66.8 (3.0) | 89  56.7 (2.9) ^**^  54.5 (3.6) ^**^ | -12.3  NA^4^ | | 573  73.5 (1.0)  66.8 (3.0) | 64  53.3 (3.5) ^**^  51.2 (3.9) ^**^ | -15.6  NA^4^ | | 623  71.8 (1.0)  64.3 (3.0) | 15  56.1 (8.0) ^ns^  58.3 (7.2) ^ns^ | -6.0  NA^4^ | |
| CF, n | | 551  77.1 (1.0)  67.5 (3.1) | 89  61.6 (3.1) ^**^  58.4 (3.7) ^*^ | -9.1  Medium | | 573  77.1 (1.0)  68.5 (3.0) | 64  55.0 (3.9) ^**^  52.0 (4.0) ^**^ | -16.5  Large | | 624  75.5 (1.0)  65.8 (3.1) | 15  55.6 (8.9) ^ns^  59.1 (7.3) ^ns^ | -6.7  Small | |
| SF, n | | 551  79.2 (1.0)  73.4 (3.2) | 89  54.3 (3.3) ^**^  54.1 (3.9) ^**^ | -19.3  Large | | 573  78.8 (1.0)  73.5 (3.2) | 64  48.4 (3.7) ^**^  49.1 (4.1) ^**^ | -24.4  Large | | 624  76.5 (1.0)  70.4 (3.2) | 15  43.3 (8.3) ^**^  48.8 (7.8) ^*^ | -21.6  Large | |
| FI, n | | 551  8.4 (0.9)  24.1 (2.7) | 89  23.2 (3.7) ^**^  31.6 (3.3) ^*^ | +7.5  Small | | 573  8.0 (0.9)  22.7 (2.6) | 64  31.8 (4.5) ^**^  38.9 (3.4) ^**^ | +16.2  Medium | | 624  9.8 (0.9)  24.8 (2.7) | 15  35.6 (10.5) ^ns^  39.1 (6.4) ^ns^ | +14.3  Medium | |

|  | | Cramps in hands | | | | Cramps in feet | | | Difficulty manipulating small objects with fingers | | | Difficulty opening a jar or bottle because of weakness in hands | | |
| --- | --- | --- | --- | --- | --- | --- | --- | --- | --- | --- | --- | --- | --- | --- |
| Scale | **Not at all-**  **A little**  Unadjusted^1^  Adjusted^2^  Mean (SE) | | **Moderate-**  **Severe**  Unadjusted^1^  Adjusted^2^  Mean (SE) | Δ^3^  ClD^4^ | **Not at all-**  **A little**  Unadjusted^1^  Adjusted^2^  Mean (SE) | | **Moderate-**  **Severe**  Unadjusted^1^  Adjusted^2^  Mean (SE) | Δ^3^  ClD^4^ | **Not at all-**  **A little**  Unadjusted^1^  Adjusted^2^  Mean (SE) | **Moderate-**  **Severe**  Unadjusted^1^  Adjusted^2^  Mean (SE) | Δ^3^  ClD^4^ | **Not at all-**  **A little**  Unadjusted^1^  Adjusted^2^  Mean (SE) | **Moderate-**  **Severe**  Unadjusted^1^  Adjusted^2^  Mean (SE) | Δ^3^  ClD^4^ |
| GHS, no | 584  70.4 (0.8)  59.4 (2.6) | | 53  52.2 (3.6) ^**^  48.8 (3.5) ^**^ | -10.6  Medium | 476  71.5 (0.9)  59.7 (2.6) | | 165  61.1 (1.7) ^**^  53.2 (2.8) ^**^ | -6.5  Small | 558  70.6 (0.9)  59.3 (2.5) | 83  56.3 (2.3) ^**^  48.4 (3.2) ^**^ | -10.9  Medium | 488  72.5 (0.9)  60.9 (2.5) | 154  57.3 (1.6) ^**^  49.4 (2.8) ^**^ | -11.5  Medium |
| PF, no | 584  84.1 (0.7)  74.9 (2.0) | | 53  64.6 (3.3) ^**^  64.4 (2.7) ^**^ | 10.5  Small | 476  85.3 (0.8)  75.6 (2.0) | | 165  73.9 (1.7) ^**^  68.2 (2.1) ^**^ | -7.4  Small | 558  84.6 (0.7)  75.2 (1.9) | 83  67.5 (2.5) ^**^  62.2 (2.4) ^**^ | -13.2  Small | 488  86.8 (0.7)  77.3 (1.9) | 154  68.7 (1.7) ^**^  63.2 (2.1) ^**^ | -14.1  Small |
| RF, no | 582  78.8 (1.1)  69.1 (3.2) | | 53  55.7 (4.1) ^**^  52.9 (4.3) ^**^ | -16.2  Small | 473  80.5 (1.2)  69.2 (3.2) | | 166  66.0 (2.2) ^**^  60.2 (3.4) ^**^ | -9.0  Small | 556  79.7 (1.1)  69.2 (3.1) | 83  57.0 (3.0) ^**^  51.4 (3.9) ^**^ | -17.8  Small | 485  83.3 (1.0)  73.3 (3.0) | 155  56.6 (2.2) ^**^  50.0 (3.3) ^**^ | -23.3  Medium |
| EF, no | 584  72.9 (1.0)  66.1 (3.0) | | 53  60.0 (4.0) ^**^  53.9 (4.1) ^**^ | -12.2  NA^4^ | 474  75.8 (1.0)  68.7 (3.0) | | 166  58.9 (2.1) ^**^  55.2 (3.2) ^**^ | -13.5  NA^4^ | 557  73.4 (1.0)  66.2 (3.0) | 83  57.6 (3.1) ^**^  52.4 (3.8) ^**^ | -13.8  NA^4^ | 486  75.9 (1.0)  68.9 (2.9) | 155  57.5 (2.2) ^**^  52.4 (3.2) ^**^ | -16.5  NA^4^ |
| CF, no | 584  76.3 (1.0)  66.8 (3.1) | | 53  61.3 (4.3) ^**^  58.3 (4.2) ^ns^ | -8.5  Small | 475  78.8 (1.1)  68.8 (3.1) | | 166  64.1 (2.3) ^**^  59.2 (3.3) ^**^ | -9.6  Medium | 558  77.5 (1.0)  68.2 (3.0) | 83  58.4 (3.4) ^**^  51.9 (3.8) ^**^ | -16.3  Large | 487  80.2 (1.0)  70.8 (2.9) | 155  59.0 (2.4) ^**^  52.9 (3.2) ^**^ | -17.9  Large |
| SF, no | 584  77.5 (1.1)  71.4 (3.3) | | 53  56.9 (4.0) ^**^  57.0 (4.5) ^**^ | -14.4  Medium | 475  80.6 (1.1)  73.7 (3.3) | | 166  62.0 (2.3) ^**^  60.1 (3.5) ^**^ | -13.6  Medium | 558  78.5 (1.1)  72.0 (3.2) | 83  57.4 (3.0) ^**^  53.9 (4.0) ^**^ | -18.1  Large | 487  81.3 (1.1)  74.5 (3.2) | 155  58.9 (2.2) ^**^  56.0 (3.5) ^**^ | -18.5  Large |
| FI, no | 584  9.0 (0.9)  23.6 (2.7) | | 53  25.8 (5.1) ^*^  35.6 (3.7) ^**^ | +12.0  Medium | 475  7.5 (0.9)  23.5 (2.7) | | 166  18.7 (2.5) ^**^  30.0 (2.9) ^**^ | +6.5  Small | 558  9.0 (1.0)  24.6 (2.7) | 83  19.7 (3.5) ^*^  31.6 (3.4) ^*^ | +7.0  Small | 487  7.8 (0.9)  23.7 (2.7) | 155  18.5 (2.5) ^**^  30.6 (3.0) ^**^ | +6.9  Small |

|  | Difficulty walking  because of foot drop | | | Difficulty climbing stairs or getting up/out of chair because of weakness in legs | | | |
| --- | --- | --- | --- | --- | --- | --- | --- |
| Scale | **Not at all**  **A little**  Unadjusted^1^  Adjusted^2^  Mean (SE) | **Moderate-**  **Severe**  Unadjusted^1^  Adjusted^2^  Mean (SE) | Δ^3^  ClD^4^ | | **Not at all**  **A little**  Unadjusted^1^  Adjusted^2^  Mean (SE) | **Moderate-**  **Severe**  Unadjusted^1^  Adjusted^2^  Mean (SE) | Δ^3^  ClD^4^ |
| GHS, no | 624  69.6 (0.8)  57.7 (2.5) | 14  41.7 (6.0) ^**^  42.9 (6.1) ^ns^ | -14.8  Medium | | 551  71.7 (0.8)  60.3 (2.5) | 92  51.4 (2.3) ^**^  45.3 (3.1) ^**^ | -15.0  Large |
| PF, no | 624  83.4 (0.7)  73.5 (2.0) | 14  45.7 (7.7) ^**^  52.4 (4.7) ^**^ | -21.1  Medium | | 551  86.2 (0.6)  76.6 (1.8) | 92  60.0 (2.3) ^**^  57.7 (2.3) ^**^ | -18.9  Medium |
| RF, no | 622  77.6 (1.0)  66.4 (3.1) | 14  40.5 (7.8) ^**^  50.1 (7.6) ^ns^ | -16.3  Small | | 548  81.2 (1.0)  70.7 (3.0) | 93  50.7 (2.9) ^**^  46.7 (3.7) ^**^ | -24.0  Medium |
| EF, no | 624  71.8 (1.0)  63.9 (3.0) | 14  53.0 (6.5) ^ns^  55.3 (7.2) ^ns^ | -8.6  NA^4^ | | 549  74.0 (1.0)  67.0 (2.9) | 93  56.0 (2.5) ^**^  50.4 (3.6) ^**^ | -16.6  NA^4^ |
| CF, no | 624  75.4 (1.0)  65.3 (3.1) | 14  59.5 (7.4) ^ns^  63.0 (7.3) ^ns^ | -2.3  Trivial | | 550  77.4 (1.0)  68.3 (3.0) | 93  60.7 (3.0) ^**^  52.7 (3.7) ^**^ | -15.6  Large |
| SF, no | 624  76.9 (1.0)  69.6 (3.2) | 14  32.1 (5.7) ^**^  36.2 (7.7) ^**^ | -33.4  Large | | 550  79.5 (1.0)  73.1 (3.1) | 93  54.3 (2.9) ^**^  50.8 (3.9) ^**^ | -22.3  Large |
| FI, no | 624  9.2 (0.9)  24.9 (2.6) | 14  54.8(11.4) ^*^  60.5 (6.2) ^**^ | +35.6  Medium | | 550  8.1 (0.9)  23.9 (2.7) | 93  23.7 (3.6) ^**^  34.0 (3.2) ^**^ | +10.1  Medium |

Abbreviations: CID, clinical important difference; GHS, Global Health Status; PF, physical functioning; RF, role functioning; EF, emotional functioning; CF, cognitive functioning, SF, social functioning; FI, financial difficulties due to the problem; NA, not applicable.

Higher scores on the QLQ-C30 global and functional scales indicate that the respondent has good general health and functioning. In contrast, a high score on FI indicates worse financial difficulties due to the problem.

^1^Student´s t-test. ** = P <0.01, *= P <0.05, ns = non-significant. The Bonferroni method was used to adjust for multiple comparisons within each symptom.

^2^Linear regression (ANCOVA) adjusted for age, BMI, civil status, educational level, employment status, alcohol consumption, exercise, smoking, musculoskeletal disorders, cardiovascular disease, diabetes mellitus, pulmonary disease, and neurological disease. ** = P <0.01, *= P <0.05, ns = non-significant. The Bonferroni method was used to adjust for multiple comparisons within each symptom.

^3^ Δ = the difference of the adjusted mean scores between those with moderate-severe and those without or a little peripheral neuropathy.

^4^ The guidelines reported by Cocks et al 2011 [11] were used to interpret the difference in mean scores. CID was categorized into four groups: a large difference was defined as one representing unequivocal clinical relevance; a median difference as clinically relevant but to a lesser extent; a small difference as clinically relevant but subtle; and a trivial difference as circumstances unlikely to have any clinical relevance. The emotional functioning subscale was omitted in the guidelines.
