# Supplementary material for: Impact of persistent peripheral neuropathy on health-related quality of life among early-stage breast cancer survivors: a population-based cross-sectional study
Source: Breast Cancer Res Treat. 2022 Aug 9;195(3):379–91. doi: 10.1007/s10549-022-06670-9 (PMC9464756; doi:10.1007/s10549-022-06670-9)
Supplement: Supplementary file 2 — Supplementary file2 (DOCX 22 kb) [file 10549_2022_6670_MOESM2_ESM.docx]

**Table A.** Basic descriptive statistics presenting the number of responses for Global Health Status/QoL, functional health **scales** and personal finances (EORTC QLQC30) and taxane-induced peripheral neuropathy (TIPN) symptoms (EORTC CIPN20). In total, 646 Early-stage breast cancer survivors (ESBCS) were included in the study and analysis.

|  | **Number of ESBCS with a TIPN symptom responding to an item in QLQ-C30** | | | | | | |
| --- | --- | --- | --- | --- | --- | --- | --- |
|  | **GHS/QoL**  **n**  **(%)** | **PF**  **n**  **(%)** | **RF**  **n**  **(%)** | **EF**  **n**  **(%)** | **CF**  **n**  **(%)** | **SF**  **n**  **(%)** | **FI**  **n**  **(%)** |
| **Tingling fingers/hands**  N, responses n=642  no. missing responses n=4 | 641 (99.8) | 641  (99.8) | 638  (99.4) | 640  (99.7) | 641  (99.8) | 641  (99.8) | 641  (99.8) |
| **Tingling toes/feet**  N, responses n=642  no. missing responses n=4 | 641  (99.8) | 641  (99.8) | 636  (99.1) | 640  (99.7) | 641  (99.8) | 641  (99.8) | 641  (99.8) |
| **Numbness fingers/hands**  N, responses n=640  no. missing responses n=6 | 639  (99.8) | 639  (99.8) | 637  (99.5) | 639  (99.8) | 639  (99.8) | 639  (99.8) | 639  (99.8) |
| **Numbness toes/feet**  N, responses n=641  no. missing responses n=5 | 640  (99.8) | 640  (99.8) | 638  (99.5) | 639  (99.7) | 640  (99.8) | 640  (99.8) | 640  (99.8) |
| **Shooting/burning in feet**  N, responses n=641  no. missing responses n=5 | 640  (99.8) | 640  (99.8) | 638  (99.5) | 639  (99.7) | 640  (99.8) | 640  (99.8) | 640  (99.8) |
| **Problems standing/walking because of difficulty feeling ground under feet**  N, responses n=638  no. missing responses n=8 | 637  (99.8) | 637  (99.8) | 635  (99.5) | 637  (99.8) | 637  (99.8) | 637  (99.8) | 637  (99.8) |
| **Difficulty distinguishing between hot/cold water**  N, responses n=640  no. missing responses n=6 | 639  (99.8) | 639  (99.8) | 637  (99.5) | 638  (99.7) | 639  (99.8) | 639  (99.8) | 639  (99.8) |
| **Cramps in hands**  N, responses n=638  no. missing responses n=8 | 637  (99.8) | 637  (99.8) | 635  (99.5) | 637  (99.8) | 637  (99.8) | 637  (99.8) | 637  (99.8) |
| **Cramps in feet**  N, responses n=642  no. missing responses n=4 | 641  (99.8) | 641  (99.8) | 639  (99.5) | 640  (99.7) | 641  (99.8) | 641  (99.8) | 641  (99.8) |
| **Difficulty manipulating small objects with fingers**  N, responses n=642  no. missing responses n=4 | 641  (99.8) | 641  (99.8) | 639  (99.5) | 640  (99.7) | 641  (99.8) | 641  (99.8) | 641  (99.8) |
| **Difficulty opening a jar or bottle because of weakness in hands**  N, responses n=643  no. missing responses n=3 | 642  (99.8) | 642  (99.8) | 640  (99.5) | 641  (99.7) | 642  (99.8) | 642  (99.8) | 642  (99.8) |
| **Difficulty walking because of foot drop**  N, responses n=639  no. missing responses n=7 | 638  (99.8) | 638  (99.8) | 636  (99.5) | 638  (99.8) | 638  (99.8) | 638  (99.8) | 638  (99.8) |
| **Difficulty climbing stairs or getting up/out of chair because of weakness in legs**  N, responses n=644  no. missing responses n=2 | 641  (99.5) | 641  (99.5) | 639  (99.2) | 640  (99.4) | 641  (99.5) | 641  (99.5) | 641  (99.5) |
